# An In Vitro Confocal Study to Compare the Effect of Two Cross-Linking Agents on Apical Microleakage of a Root-End Filling Material

**DOI:** 10.7759/cureus.97233

**Published:** 2025-11-19

**Authors:** Harpreet Chhabra, Ikshita Deshwal, Sanjeev Srivastava, Kshiti Chhabra, Aditya Singh, Preeti Rastogi

**Affiliations:** 1 Department of Conservative Dentistry and Endodontics, Sardar Patel Post Graduate Institute of Dental & Medical Sciences, Lucknow, IND

**Keywords:** apical microleakage, bioactive glass, mineral trioxide aggregate, periradicular surgery, proanthocyanidin

## Abstract

Objective(s): This in vitro study was conducted to assess the impact of bioactive glass (BAG) and 6.5% proanthocyanidin (PA) as pretreatment agents on apical microleakage of the root end filling material.

Materials and methods: Forty-five single-rooted premolars were cleaned, autoclaved, and underwent root canal treatment. The teeth were decoronated to standardize tooth length to 14 mm, with the coronal end sealed with flowable composite. Apical resection at 3 mm from the apex was performed, followed by root end cavity preparation using ultrasonic tips to a depth of 3 mm. Samples were then divided into three groups (n=15): Group A (no pretreatment before mineral trioxide aggregate (MTA) placement), Group B (6.5% PA pretreatment before MTA placement), and Group C (bioactive glass pretreatment before MTA placement). After pretreatment and root end filling in respective groups, samples were coated with nail varnish (except the surface restored with MTA) and immersed in 2% rhodamine B dye for 24 hours, followed by longitudinal sectioning, and examined for apical dye penetration under CLSM (confocal laser scanning microscope) at 40X magnification.

Results: Comparing the mean dye penetration of three groups, ANOVA showed statistically significant dye penetration among the groups (F=117.40, p < 0.001). Further, comparing the difference in mean dye penetration between the groups, Tukey's post-hoc test showed significantly lower dye penetration in both Group B and Group C as compared to Group A. Moreover, the difference in mean dye penetration of Group C showed significantly lower dye penetration as compared to Group B.

Conclusion(s): Pretreatment agents significantly improved the sealing ability of root-end filling material by enhancing bonding to dentin through collagen cross-linking, which stabilizes exposed collagen and promotes mineral deposition, thus preventing bacterial infiltration over time.

## Introduction

Conventional endodontic treatment focuses on eradicating bacteria and achieving an impervious seal to prevent recontamination. Successful treatment requires thorough cleaning, shaping, and sealing of the root canal system. If the apical seal is compromised, microorganisms and toxins can travel through the root apex, lateral canals, and apical ramifications to the periapical region, leading to reinfection [1].

While nonsurgical treatments are often effective, surgery may become necessary for persistent periradicular pathosis. Moreover, in situations where the apical seal cannot be achieved, such as calcified canals, canals with an open apex, complex apical ramifications, or root resorption, root-end resection followed by root-end filling is required [2]. Root-end filling is essential in such cases to form a fluid-tight apical barrier and prevent the migration of bacteria and toxins into the periapex. An ideal root-end filling material should be biocompatible, dimensionally stable, and able to bond securely with the prepared cavity walls. Materials such as mineral trioxide aggregate (MTA) have been considered superior due to their high pH, osteoinductive properties, and ability to set in the presence of body fluids [3].

During root canal therapy, irrigation plays a vital role in eliminating debris and microorganisms while disinfecting the canal system. Sodium hypochlorite (NaOCl), regarded as the gold standard irrigant, combines potent antimicrobial activity with the ability to dissolve organic tissue, though its proteolytic effect may degrade dentinal collagen and compromise dentin integrity [4,5]. To complement NaOCl, ethylenediaminetetraacetic acid (EDTA) is used to remove the inorganic component of the smear layer through calcium ion chelation, thereby enhancing dentinal tubule exposure and irrigant penetration [6]. However, the sequential use of NaOCl and EDTA may adversely alter dentin by promoting collagen degradation and mineral loss, leading to surface softening and potential erosion that can affect the quality of root-end preparation and long-term sealing [7].

To counteract this, pretreatment with cross-linking and remineralizing agents has been suggested. Cross-linking agents reinforce the collagen network by forming additional covalent bonds between collagen fibers, while remineralizing agents restore lost minerals, especially hydroxyapatite, by reintroducing calcium and phosphate ions into the dentin [8]. Recently, various agents have been used, among which proanthocyanidin (PA) and bioactive glass (BAG) have become common pretreatment agents.

PA is a crosslinking agent that strengthens dentin by promoting collagen cross-linking, increasing resistance to collagenase degradation, and inhibiting matrix metalloproteinases (MMPs). This enhances dentin collagen stability and restoration durability while promoting mineral penetration, thereby improving the strength and elasticity of demineralized dentin [9]. BAG, on the other hand, is a pretreatment agent with osteoconductive, osteoinductive, and biocompatible properties. It releases calcium and silica ions that support remineralization and hydroxyapatite formation while inhibiting MMPs to protect the dentin matrix from collagen degradation. Additionally, BAG exhibits antibacterial properties, as the release of calcium, phosphate, and silica ions disrupts bacterial metabolism, preserving restoration bonding and preventing material degradation caused by bacterial infiltration [10].

However, the efficacy of these pretreatment agents in preventing microleakage prior to root-end cavity restoration remains unclear. Thus, this research aimed to assess their impact on apical microleakage, with the goal of improving long-term treatment success. Although clinically undetectable, microleakage allows bacteria, fluids, and debris to enter, potentially causing reinfection and treatment failure. Therefore, investigating the effectiveness of these agents in preventing microleakage is crucial.

## Materials and methods

A total of 45 permanent single-rooted mandibular premolars, extracted for therapeutic purposes within one month in August 2024, were obtained from the Department of Oral and Maxillofacial Surgery, Sardar Patel Postgraduate Institute of Dental and Medical Sciences, Lucknow, India. The extracted teeth were cleaned under running water, and all adherent stains and calculus were removed using an ultrasonic scaler. Each specimen was examined radiographically to confirm eligibility. Fully developed, single-rooted, non-restored teeth with straight roots and a single canal were included in the study, while grossly decayed teeth, previously restored teeth, and those with developmental anomalies or fractures were excluded. Following selection, the teeth were autoclaved at 121°C and 15 psi for 15 minutes and stored in distilled water until further use.

Root Canal Preparation

Standard endodontic access cavities were prepared using a high-speed handpiece and diamond burs. Working length was determined using a No. 15 K-file inserted until the tip was visible at the apex, then confirmed radiographically, and adjusted to 1 mm short of the apex. Root canals were shaped using ProTaper Gold rotary files up to F3 under continuous irrigation with 3% NaOCl between files, followed by a final rinse with 17% EDTA for one minute to remove the smear layer. Canals were then dried with sterile paper points and obturated using the single-cone technique with F3 gutta-percha cones and AH-Plus sealer.

**Figure 1:**
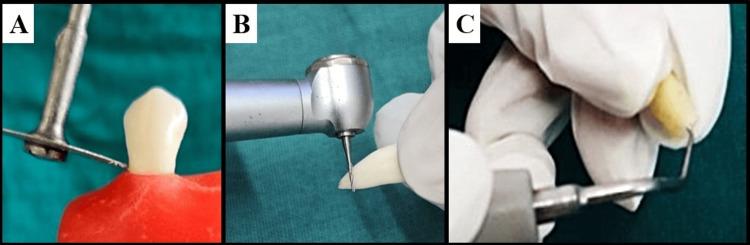
Tooth preparation A - Decoronating the obturated tooth to standardize the root length to 14 mm. B - 3 mm of root-end resection using a carbide fissure bur. C - Root-end cavity preparation using an ultrasonic tip

Each specimen was decoronated at the cementoenamel junction (CEJ) using a diamond disk to standardize root length to 14 mm (Figure 1A). The coronal 3 mm of the obturated material was removed and sealed with flowable composite resin to prevent coronal leakage.

Apical Resection and Root-End Cavity Preparation

After obturation, samples were stored in 100% humidity at 37°C for one week to allow complete sealer setting and simulate intraoral conditions. A 3 mm apical resection was then performed using a carbide fissure bur under water coolant at high speed (Figure 1B), reducing approximately 98% of apical ramifications and 93% of lateral canals. This procedure minimized the influence of anatomical irregularities. Subsequently, 3 mm deep root-end cavities were prepared using ultrasonic retro-tips to achieve standardized dimensions and ensure adequate bulk for retrofilling (Figure 1C).

Despite these measures, it must be acknowledged that the in vitro conditions cannot fully replicate the complex intraoral environment, where variables such as saliva, temperature fluctuations, masticatory forces, and pH variations influence material behavior, sealing ability, and longevity.

Pretreatment of Root-End Cavity With Bioactive Glass and 6.5% Proanthocyanidin

A 20% bioactive glass slurry (45S5) was prepared by adding 20 g of bioactive glass powder to 100 mL of distilled water and stirring until homogeneous. A 6.5% proanthocyanidin solution was prepared by dissolving 6.5 g of grape seed extract (proanthocyanidin) in 100 mL of distilled water.

The teeth were randomly divided into three groups (n = 15 each):

Group A: MTA was placed in the root-end cavity without pretreatment.

Group B: Root-end cavity pretreated with 6.5% proanthocyanidin for five minutes, rinsed with distilled water, and air-dried, followed by MTA placement.

Group C: Root-end cavity pretreated with 20% bioactive glass slurry for five minutes, rinsed, and air-dried, followed by MTA placement.

**Figure 2:**
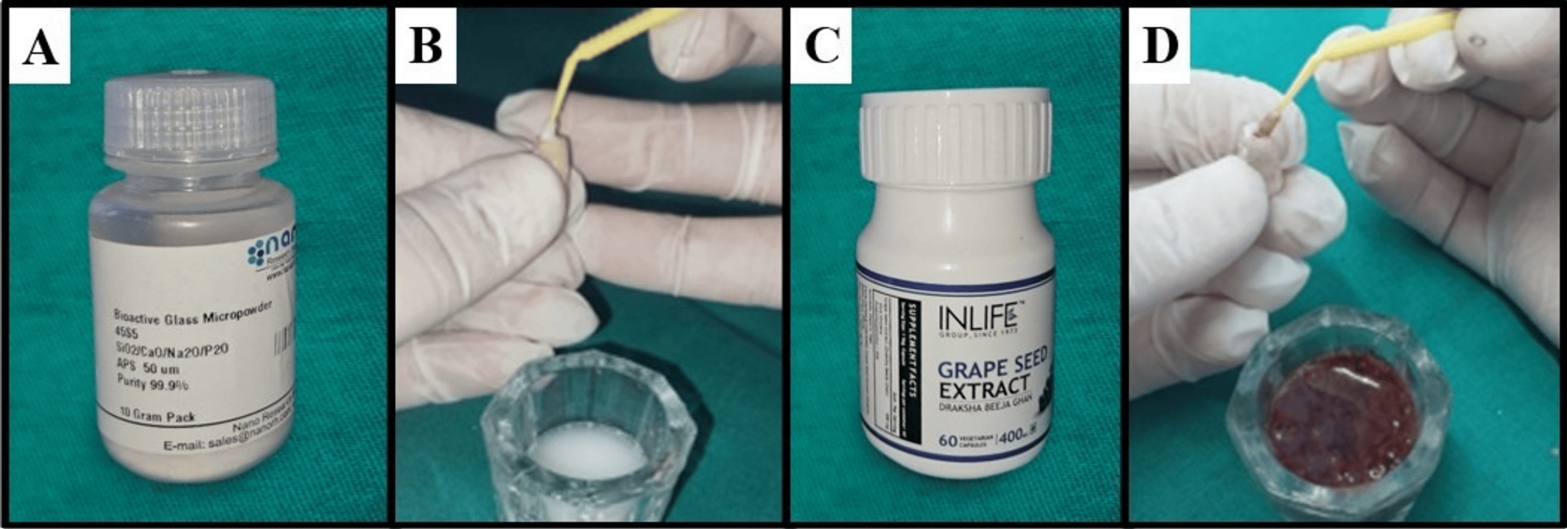
Pretreatment of root-end cavity A - Bioactive glass powder. B - Pretreatment of root-end cavity with bioactive glass slurry. C - Proanthocyanidin (grape seed extract). D - Pretreatment of root-end cavity with 6.5% proanthocyanidin A and B represent pretreatment with bioactive glass (BAG), while C and D correspond to pretreatment with 6.5% proanthocyanidin.

**Figure 3:**
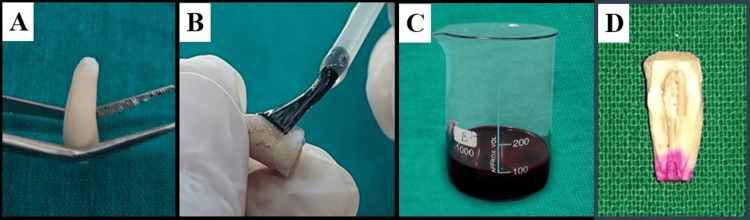
Root end cavity sealing, followed by dye immersion and longitudinal sectioning A - Root end cavity restored with mineral trioxide aggregate (MTA). B - Application of nail varnish over the samples, leaving the restored root-end cavity exposed. C - Immersion of samples in 2% Rhodamine B dye solution for 24 hours. D - Longitudinal sectioning of the sample

After pretreatment (Figures 2A-2D) and root-end filling with MTA (Figure 3A), samples were allowed to set for 24 hours at 37°C in 100% humidity. Then, two layers of nail varnish were applied to the entire root surface, except for the prepared cavity, to prevent extraneous dye penetration (Figure 3B).

After drying for one hour, the roots were immersed in 2% Rhodamine B dye for 24 hours (Figure 3C). The samples were then sectioned longitudinally in the buccolingual direction using a diamond disk under water coolant to obtain two halves (Figure 3D).

**Figure 4:**
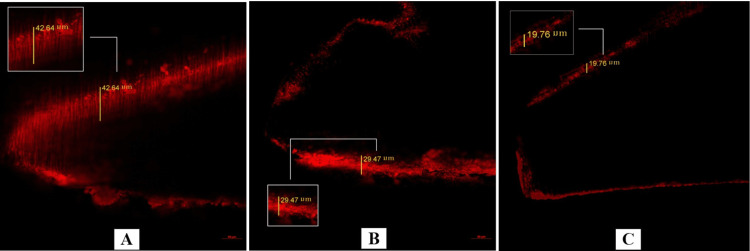
Confocal laser scanning microscope (CLSM) images of the samples at 40x magnification A - CLSM image of apical dye penetration depth in Group A. B - CLSM image of apical dye penetration depth in Group B. C - CLSM image of apical dye penetration depth in Group C

All specimens were examined under a Confocal Laser Scanning Microscope (CLSM) at 40× magnification to measure the depth of dye penetration in micrometers (Figures 4A-4C). The recorded values were tabulated and subjected to statistical analysis.

Statistical Analysis

The data were expressed as mean ± standard error (SE). The normality of distribution was assessed using the Shapiro-Wilk test, and homogeneity of variances was verified by Levene’s test. Intergroup comparison was carried out using one-way analysis of variance (ANOVA). When significant differences were detected, Tukey’s honestly significant difference (HSD) post-hoc test was applied to determine pairwise differences. A two-tailed p value < 0.05 was considered statistically significant. All statistical analyses were performed using Statistical Product and Service Solutions (SPSS, version 22.0; IBM SPSS Statistics for Windows, Armonk, NY) software.

## Results

The dye penetration values for the three experimental groups are summarized in Table 1.

**Table 1:** Dye penetration (µm) of the three groups Dye penetration of the three groups was summarised in range (min to max), Mean ± SE and median.

Group	n	Min to Max	Mean ± SE	Median
Group A	15	40.91 to 73.29	53.95 ± 2.84	51
Group B	15	22.10 to 34.09	28.04 ± 1.01	27
Group C	15	11.25 to 21.11	15.65 ± 0.83	15

Group A exhibited dye penetration ranging from 40.91 µm to 73.29 µm, with a mean ± SE of 53.95 ± 2.84 µm and a median value of 51 µm. Group B showed a range of 22.10 µm to 34.09 µm, with a mean ± SE of 28.04 ± 1.01 µm and a median of 27 µm. Group C demonstrated the lowest values, ranging from 11.25 µm to 21.11 µm, with a mean ± SE of 15.65 ± 0.83 µm and a median of 15 µm.

Among the groups, Group C had the lowest mean dye penetration, followed by Group B, while Group A showed the highest values (Group C < Group B < Group A).

ANOVA revealed a statistically significant difference in mean dye penetration among the three groups (F = 117.40; p < 0.001) (Table 2).

**Table 2:** Comparison of mean dye penetration (µm) of the three groups by analysis of variance (ANOVA) F value: ANOVA F value

Source of variation (SV)	Sum of square (SS)	Degree of freedom (DF)	Mean square (MS)	F value	P value
Groups	11460.00	2	5730.00	117.40	< 0.001
Residual	2050.00	42	48.82		
Total	13510.00	44	5778.82		

Post hoc analysis using Tukey's HSD test demonstrated that the mean dye penetration in Group B and Group C was significantly lower than that in Group A (p < 0.001 for both comparisons). Additionally, Group C showed a significantly lower mean dye penetration than Group B (p < 0.001) (Table 3).

**Table 3:** Comparison of differences in mean dye penetration (µm) between the groups by Tukey's test CI: confidence interval, diff: difference, q value: Tukey's test value

Comparison	Mean diff.	q value	P value	95% CI of diff.
Group A vs. Group B	25.91	14.36	P < 0.001	19.71 to 32.11
Group A vs. Group C	38.30	21.23	P < 0.001	32.10 to 44.50
Group B vs. Group C	12.39	6.87	P < 0.001	6.19 to 18.59

In terms of percentage reduction, the mean dye penetration in Group C was 71.0% lower than Group A and 44.2% lower than Group B. Group B also exhibited a 48.0% reduction in mean dye penetration compared to Group A.

## Discussion

While conventional endodontic treatment boasts a success rate of up to 90-95%, failures still occur in 5-10% of cases. These failures are often attributed to persistent infection, anatomical complexities, cysts or abscesses, and root resorption. The leaching of remanent bacteria or their byproducts into the periapical region can jeopardize the outcome of non-surgical endodontic treatment by activating host immune responses and sustaining or reinitiating periapical inflammation. Therefore, a proper apical seal is required for the long-term prognosis of the treatment. However, when non-surgical treatment is not possible (anatomical complexities or calcified canals) or if the symptoms persist, endodontic surgery becomes necessary to save the tooth [11]. In such cases, root-end resection becomes necessary, which requires additional root-end filling materials to ensure a proper apical seal.

While calcium silicate-based materials such as MTA and biodentine are commonly used for their osteoinductive and osteoconductive properties, they do not chemically bond to tooth structure and require adjunctive procedures, such as pretreatment of the root canal dentin for effective sealing. Motwani et al. [12] emphasized that the complex structure of dentin makes it challenging to achieve effective bonding with various restorative materials. They suggested that pretreating dentin with crosslinking or remineralizing agents could enhance this bond.

In the present study, pretreatment with PA and bioactive glass effectively reduced dye penetration. Group A (root-end filling without pretreatment) showed the maximum mean dye penetration depth at 53.95 ± 2.84 µm. Group B (pretreatment with PA) had 28.04 ± 1.01 µm, while Group C (pretreatment with bioactive glass) exhibited the lowest dye penetration at 15.65 ± 0.83 µm. This difference indicates that the pretreatment agents improved the adhesion between MTA and dentin in the root-end cavity, likely through collagen crosslinking and the formation of hydroxyapatite (HAP) structures. These processes contributed to the stabilization of the restoration and helped prevent apical microleakage.

Group A (no pretreatment) showed the maximum apical microleakage. MTA, despite its biological advantages, was less effective in sealing compared to the experimental groups. This was due to handling difficulties, extended setting time, and susceptibility to disintegration, which resulted in cement leaching and bacterial leakage. A study done by Abbas et al. [13] found that contamination of unset MTA with tissue fluids deteriorates its mechanical properties, while Nekoofar et al. [14] also noted that such contamination alters MTA's setting reaction, thus reducing the formation of calcium hydroxide crystalline structures. Therefore, exploring additives and alternatives is essential to enhance MTA’s performance.

Group B, with PA pretreatment, showed less apical microleakage than Group A (no pretreatment). PA forms insoluble complexes with collagen through hydrogen bonding, covalent, and hydrophobic interactions, stabilizing collagen and enhancing its mechanical properties. Verma et al. [15] found that PA stabilizes dentin collagen and improves resistance to biodegradation. It also accelerates collagen conversion and enhances mineral deposition, as noted by Benjamin et al. [16]. When PA is used to support MTA adhesion to dentin, the quality and durability of the bond between MTA and dentin improve, leading to better clinical outcomes. Collagen stabilization enhances bond strength and durability by providing a more stable and resilient bonding substrate for MTA. PA also contributes to the remineralization process by facilitating the deposition of calcium and phosphate ions onto collagen fibrils, thereby promoting the nucleation and growth of HAP crystals. A similar study was conducted by Epasinghe et al. [17], which investigated the effects of PA on ultrastructure and mineralization of dentin collagen and found that treatment with 6.5% PA reduced interfibrillar spaces and enhanced mineral aggregation along the collagen fibrils.

Group C, where BAG was used as a pretreatment agent, showed the least apical microleakage. BAG interacts with collagen fibers, forming a HAP layer that occludes dentinal tubules and resists acidic environments. Additionally, its reactivity creates a negative surface charge, which binds to type I collagen fibers in dentin. Skallevold et al. [18] observed that BAG promotes HAP crystallization, sealing the dentinal tubules. BAG's superior remineralizing capacity is attributed to its higher calcium and phosphate content. Joshi et al. [19] found that BAG deposits were larger and more angular than those formed by PA, resulting in a more compact and adhesive plug. The enhanced remineralization and crosslinking with BAG improved the bonding of the restoration to root dentin. A similar study conducted by Mendoza et al. [20] evaluated the physical properties of MTA with the addition of BAG and concluded that incorporating BAG into MTA significantly reduced microleakage and improved MTA's mechanical properties. BAG also increases the pH of the surrounding environment, which enhances MTA’s antimicrobial effect. Kim et al. [21] reported improved MTA properties when combined with BAG, and Flores-Ledesma et al. [22] found that BAG reduced MTA’s setting time without compromising its biological response, enhancing mineralization and dentin bonding.

The results of the present study demonstrate that Group C (BAG pretreatment) achieved the lowest apical microleakage, followed by Group B (PA pretreatment), whereas Group A (no pretreatment) exhibited the highest leakage values. This outcome indicates that dentin pretreatment enhances the integrity of the hybrid layer by reinforcing collagen fibrils, facilitating remineralization, and improving the long-term stability of the adhesive interface. Through the promotion of hydroxyapatite deposition and improved substrate stability, these agents minimize cement dissolution and enhance the marginal sealing capacity of root-end fillings, thereby potentially reducing the risk of microleakage and bacterial recontamination.

While the data are encouraging, several limitations must be acknowledged. As an in vitro investigation, the study cannot fully simulate clinical variables such as blood contamination, occlusal loading, salivary flow, pH fluctuations, and microbial challenges. Furthermore, the dye penetration technique, though widely used, provides a semi-quantitative estimate of leakage and may not accurately represent bacterial ingress. Only a single concentration and application protocol for each agent was evaluated, limiting the generalizability of the results. Moreover, the long-term influence of these agents on periapical healing and interface stability was not examined.

Another important consideration pertains to the biological safety of the tested agents. The present study did not include cytotoxicity or biocompatibility assessments. Although bioactive glass and PA are generally regarded as biocompatible, their potential effects on periapical tissues - such as inflammatory or immunologic responses - require further in vivo verification. Future research incorporating cell culture, histological, and animal model evaluations would be instrumental in confirming their biological safety and clinical feasibility.

Additionally, the controlled laboratory setting - characterized by standardized humidity, temperature, and absence of thermal or mechanical stress - does not accurately replicate intraoral conditions. Therefore, the sealing efficacy and bond durability observed here may not directly translate to clinical performance. Operator-related variability in cavity preparation and material manipulation, along with possible sectioning or measurement artifacts, may also have introduced experimental bias.

In summary, within the constraints of this in vitro design, bioactive glass and PA pretreatments demonstrated superior sealing performance and interface adaptation compared to untreated specimens. Confocal microscopic observations revealed more uniform and deeper penetration of the sealing material in treated groups, signifying enhanced dentin-material interaction. These findings suggest that bioactive surface modification strategies can strengthen the apical seal and reduce microleakage in root-end fillings. Nevertheless, further in vivo investigations and long-term clinical trials are necessary to substantiate these findings, assess their biocompatibility, and determine their translational potential in endodontic microsurgery.

## Conclusions

Within the limitations of this in vitro study, it can be concluded that dentin pretreatment with PA and BAG significantly enhances the sealing ability of a root-end filling material. Among the tested groups, BAG pretreatment demonstrated the least apical microleakage, followed by PA, while the non-pretreated group showed the highest leakage. The improved performance of these agents can be attributed to collagen crosslinking, enhanced remineralization, and the formation of hydroxyapatite structures, which together provide a more stable and durable bonding substrate for a root-end filling material. These findings highlight the potential of dentin pretreatment strategies to improve the long-term prognosis of surgical endodontic treatment. However, further in vivo studies with varied concentrations, clinical simulations, and long-term evaluations are needed before these approaches can be recommended for routine clinical practice.
